# Enhancing Electrocatalytic Semihydrogenation of Alkynes via Weakening Alkene Adsorption over Electron-Depleted Cu Nanowires

**DOI:** 10.1021/acsnanoscienceau.4c00030

**Published:** 2024-08-08

**Authors:** Dan Luo, Zhiheng Xie, Shuangqun Chen, Tianyi Yang, Yalin Guo, Ying Liu, Zhouhao Zhu, Liyong Gan, Lingmei Liu, Jianfeng Huang

**Affiliations:** † State Key Laboratory of Coal Mine Disaster Dynamics and Control, Institute of Advanced Interdisciplinary Studies, School of Chemistry and Chemical Engineering, Chongqing University, Chongqing 400044, China; ‡ College of Physics and Center of Quantum Materials and Devices, Chongqing University, Chongqing 401331, China

**Keywords:** electrocatalysis, semihydrogenation, Cu−Ag hybrids, alkyne, alkene desorption

## Abstract

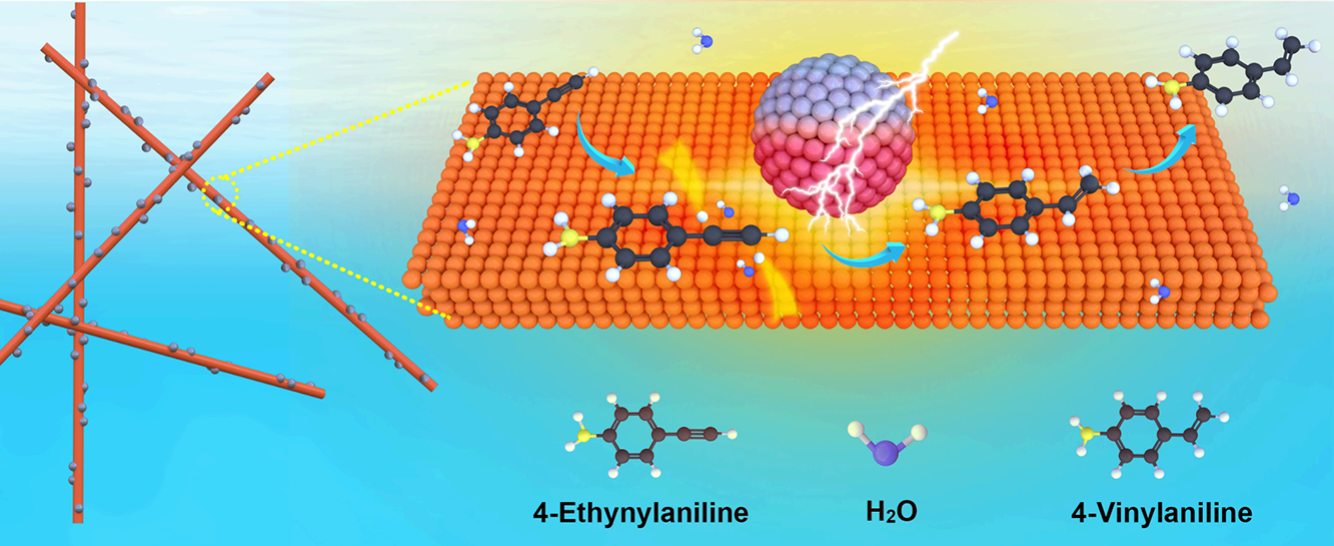

Electrochemical semihydrogenation (ESH) of alkynes to alkenes is an appealing technique for producing pharmaceutical precursors and polymer monomers, while also preventing catalyst poisoning by alkyne impurities. Cu is recognized as a cost-effective and highly selective catalyst for ESH, whereas its activity is somewhat limited. Here, from a mechanistic standpoint, we hypothesize that electron-deficient Cu can enhance ESH activity by promoting the rate-determining step of alkene desorption. We test this hypothesis by utilizing Cu–Ag hybrids as electrocatalysts, developed through a welding process of Ag nanoparticles with Cu nanowires. Our findings reveal that these rationally engineered Cu–Ag hybrids exhibit a notable enhancement (2–4 times greater) in alkyne conversion rates compared to isolated Ag NPs or Cu NWs, while maintaining over 99% selectivity for alkene products. Through a combination of operando and computational studies, we verify that the electron-depleted Cu sites, resulting from electron transfer between Ag nanoparticles and Cu nanowires, effectively weaken the adsorption of alkenes, thereby substantially boosting ESH activity. This work not only provides mechanistic insights into ESH but also stimulates compelling strategies involving hybridizing distinct metals to optimize ESH activity.

## Introduction

The semihydrogenation of alkynes to alkenes, avoiding further hydrogenation to alkane, is a crucial industrial process that not only produces key chemical intermediates and end products across various industries but also eliminates trace alkynes coexisting with alkenes to achieve high-purity alkenes.^1−5^ For instance, styrene, which is extensively used as an important reaction precursor in the pharmaceutical, agrochemical and material sectors, well illustrates this necessity.^6−12^ During the polymerization of styrene, the presence of excess phenylacetylene can poison the catalyst. Therefore, the selective semihydrogenation of alkynes to alkenes serves as an effective method to remove alkynes from polymer-grade alkenes, ensuring the purity and quality of the final products.^13−16^

The semihydrogenation of alkynes has conventionally been carried out through thermo-catalysis, with several processes demonstrating high selectivity and activity for phenylacetylene even at low temperatures.^17−20^ However, the widespread use of gaseous hydrogen (H_2_) or costly and/or toxic organic hydrogen sources for thermal semihydrogenation has constrained the robust development of selective hydrogenation.^21,22^ Recently, driven by the push for renewable and clean energy sources, electrochemical semihydrogenation (ESH), which uses water as a hydrogen source, has emerged as a new, environmentally friendly direction for selective hydrogenation with low energy consumption.^23−31^ Palladium (Pd), known for its effectiveness as a hydrogenation catalyst,^32,33^ has been extensively explored for ESH and has shown excellent activity.^24^ However, Pd also tends to overhydrogenate, leading to poor selectivity for alkenes. Currently, major challenges with Pd-catalyzed ESH include reliance on this precious metal and the complex modification needed to enhance alkene selectivity.^34^ Thus, there is an urgent need to develop more cost-effective and selective catalysts to replace Pd for ESH.

In the search for less noble alternatives to Pd for ESH, copper (Cu) has shown promise due to its unique electronic properties that favor moderate adsorption of key intermediates, similar to its role in electrocatalytic CO_2_ reduction.^35^ This specificity of Cu ensures both effective reactant binding for protonation and desirable target-product desorption.^36^ Typically, the ESH of alkynes involves two successive additions of H* atoms, derived from H_2_O, followed by the desorption of the alkenes. Although Cu exhibits high alkene selectivity—due to its fully valenced d-band, which generates strong repulsive forces against the antibonding orbitals of the alkenes’ C = C bond, thus protecting the alkenes from further hydrogenation^37,38^—its ESH activity could still benefit from electronic structure optimization.^14,15,39−41^ Previous studies have indicated that the desorption of alkenes is the rate-determining step during the ESH of phenylacetylene,^42^ suggesting that elevating the desorption rates could enhance the ESH kinetics. Strong binding of alkenes to the catalyst surface retards their desorption and leads to their accumulation, which, according to Le Chatelier’s principle, hinders the progression of preceding hydrogenation steps. Consequently, reducing the strength of alkene adsorption on the catalyst is crucial for enhancing ESH activity.

Our previous findings show that Cu can become positively charged through electron transfer to neighboring metals like silver (Ag), which possess higher electronegativities.^43^ This electronic interaction results in a downward shift of Cu’s d-band center from the Fermi level, weakening its bonding with adsorbates.^44−46^ Based on this, we hypothesize that modifying Cu by hybridizing it with Ag could effectively weaken alkene adsorption, thus facilitating faster desorption and overall hydrogenation kinetics. Additionally, the timely desorption of alkenes might decrease the likelihood of overhydrogenation, thereby increasing the selectivity for alkenes.

Here, to test our hypothesis, we fabricate Cu–Ag hybrids (denoted as CuNW@AgNPs) by welding as-synthesized Ag nanoparticles (Ag NPs) with Cu nanowires (Cu NWs). The resulting CuNW@AgNP hybrids exhibit the expected charge transfer between Cu and Ag, as confirmed by XPS and XAS. When tested as electrocatalysts for the ESH of 4-ethynylaniline (EYA) to 4-vinylaniline (VYA), the CuNW@AgNP hybrids with an optimized Cu/Ag atomic ratio (i.e., 96/4) demonstrate a markedly enhanced conversion rate over that of the pristine Cu NWs (∼2 times) and Ag NPs (∼3.4 times), while maintaining a high VYA selectivity of 99%. The enhanced activity is the result of promoted desorption of the alkene over the electron-depleted Cu, while the high selectivity originates from a lower desorption barrier compared to that of alkene hydrogenation, as validated by DFT calculations. Operando XAS tests further identify that the electron-deficient property of Cu in the CuNW@AgNP hybrids is well-preserved by the Ag NPs, contributing to good recyclability for up to 8 cycles.

## Results and Discussion

### Synthesis and Characterizations of CuNW@AgNP Hybrids

To prepare the CuNW@AgNP hybrids, presynthesized Ag NPs and Cu NWs dispersed in hexane were physically mixed in a predetermined ratio. This mixture was subjected to ultrasonication for homogenization, followed by vacuum drying at room temperature (details provided in the Experimental Section). By adjusting the amount of Ag NPs in the mixture, CuNW@AgNP hybrids with three different Cu/Ag atomic ratios (99.3/0.7, 96/4, 90/10) were obtained, denoted as Cu_99.3_NW@Ag_0.7_NP, Cu_96_NW@Ag_4_NP, and Cu_90_NW@Ag_10_NP hybrids, respectively.

**Figure 1:**
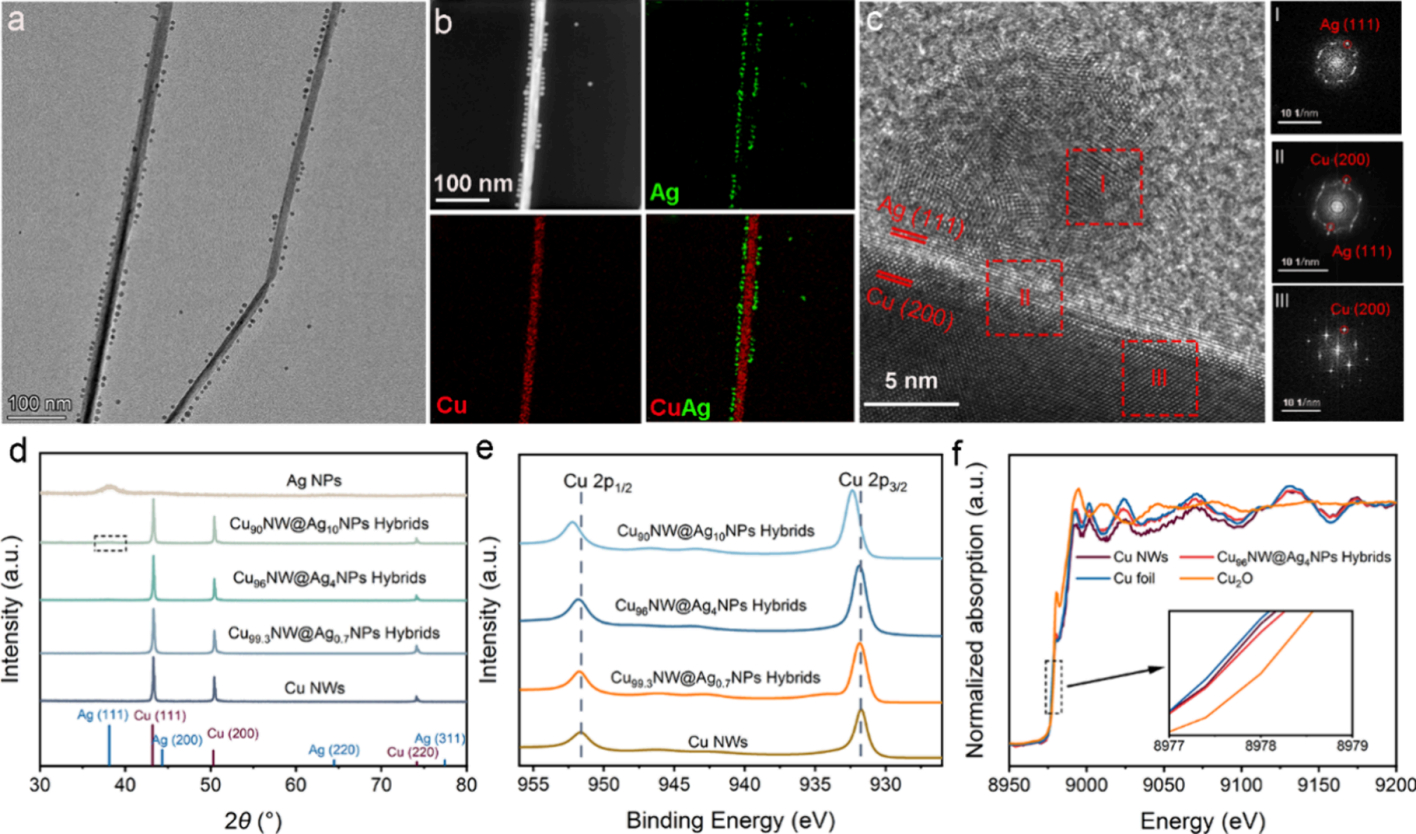
(a) TEM image of Cu_96_NW@Ag_4_NP hybrids; (b) HAADF-STEM image and the corresponding elemental mappings of one Cu_96_NW@Ag_4_NP hybrid; (c) HRTEM image of one Cu_96_NW@Ag_4_NP hybrid & FFT diffractograms of regions I–III marked; (d) XRD patterns of Ag NPs, Cu NWs, and CuNW@AgNP hybrids; the marked region indicates a diffraction peak from Ag (111) planes (see Figure S5); (e) Cu 2p XPS spectra of CuNW@AgNP hybrids and Cu NWs; (f) XANES spectra of Cu_96_NW@Ag_4_NPs and Cu NWs.

Figure S1a presents a low-magnification transmission electron microscopy (TEM) image of the pristine Cu NWs, showcasing their characteristic wire morphology with a diameter of 24.2 ± 3.0 nm and lengths spanning several micrometers (Figure S1b). The high-resolution TEM (HRTEM) image (Figure S1c) and the corresponding fast Fourier transform (FFT) pattern (Figure S1d) reveal the single-crystalline nature of the Cu NWs, identifying lattice spacing, for instance, of 0.18 nm, which is characteristic of the (200) plane of Cu. Consistent with previous studies, the Cu NWs exhibit a penta-twinned structure with sides enclosed by {100} facets.^12,47^ By contrast, the pristine Ag NPs are polycrystalline with an average size of 13.6 ± 1.0 nm (Figure S2). When Ag NPs and Cu NWs were physically mixed and vacuum-dried, the resulting CuNW@AgNP hybrids maintained the overall wire morphology, with Ag NPs linearly attached along the sides of the Cu NWs (Figures 1a and Figure S3). This arrangement is further demonstrated by energy-dispersive X-ray elemental mapping, which shows the wire and small particles composed of Cu (red) and Ag (green) elements, respectively (Figure 1b and Figure S4). Interestingly, although Ag NPs were not chemically grown on the Cu NWs, HRTEM images of the crystal structure reveal that Ag NPs are fused with Cu NWs, forming a bimetallic interface between Cu (100) facets and Ag (111) facets (Figure 1c). Such fusion of physically mixed nanoparticles has also been observed in other systems, driven by processes like wetting in Ag–Au hybrids^48^ and atom diffusion in Au-chalcogenide hybrids.^49^ While the precise mechanism behind the merging of separate Cu NWs and Ag NPs in our system remains to be fully elucidated, the successful welding provides ideal Cu–Ag hybrids to test our hypothesis.

To explore the potential influence of added Ag NPs on the crystal structure and electronic properties of the Cu NWs, we employed X-ray diffraction (XRD), X-ray photoelectron spectroscopy (XPS), and X-ray absorption spectroscopy (XAS) to characterize both Cu NWs and CuNW@AgNPs hybrids. As illustrated in Figure 1d, the Cu NWs and CuNW@AgNP hybrids display typical diffraction peaks at 43.3, 50.4, and 74.1°, corresponding to the (111), (200), and (220) planes of face-centered cubic (fcc) Cu (JCPDS 024–0836).^50^ Diffraction peaks from the Ag component were only observed when the Ag content was sufficiently high, as in Cu_90_NW@Ag_10_NPs (Figure 1d and Figure S5). Importantly, the lack of Bragg angular shifts in the CuNW@AgNP hybrids compared to pure Cu NWs indicates that the attachment of Ag NPs did not alter the Cu NWs’ crystal structure significantly. This suggests that Cu and Ag largely retain their separate phases without forming substantial Cu–Ag alloys at the interface.^43^

However, the attachment of Ag NPs significantly affected the electronic structure of the Cu NWs within the CuNW@AgNP hybrids. XPS spectra reveal that as the Ag content increased, there was a progressive positive shift in the binding energy of Cu 2p in the hybrids compared to pure Cu NWs. For example, the Cu 2p_3/2_ peak in the Cu_90_NW@Ag_10_NP hybrids shifted to 932.4 eV from 931.9 eV in Cu NWs, representing an increase of 0.5 eV (Figure 1e and Figures S6 and S7). This positive binding energy shift, driven by electron transfer from Cu to Ag, leads to the formation of electron-deficient Cu sites, which are expected to enhance the desorption of specific adsorbates. Additionally, we examined the electronic structure using XAS (Figure 1f). The Cu K-edge extended X-ray absorption near-edge structure (XANES) spectra show that both the Cu NWs and Cu_96_NW@Ag_4_NP hybrids exhibit edge absorption characteristics very similar to Cu foil, indicative of their predominantly metallic phases, as corroborated by XRD results in Figure 1d. Furthermore, their absorption was positioned between those of Cu_2_O and Cu foil, suggesting the presence of a positive valence state in the Cu NWs and Cu_96_NW@Ag_4_NP hybrids. Notably, the absorption edge of the Cu_96_NW@Ag_4_NP hybrids was slightly shifted to the right of that of Cu NWs, signaling a more positive valence for Cu in the hybrids due to the aforementioned electron donation.

### ESH Performance of CuNW@AgNP Hybrids

The performance of CuNW@AgNP hybrids in the ESH of alkynes was assessed at room temperature using an H-type three-electrode cell. The hydrogenation of 4-ethynylaniline (EYA) to 4-vinylaniline (VYA) was chosen as the proof-of-concept reaction. Catalysts supported on carbon paper, along with Pt mesh and Hg/HgO electrode were used as the working, counter, and reference electrode, respectively. The electrolyte was a 1.0 M KOH aqueous solution containing 1,4-dioxane (V_Dioxane_/V_Water_ = 2/5). All potentials were referenced to the reversible hydrogen electrode (RHE), unless otherwise stated. Products from the cathodic compartment were quantified by gas chromatography (GC), based on the calibration curves provided in Figure S8.

**Figure 2:**
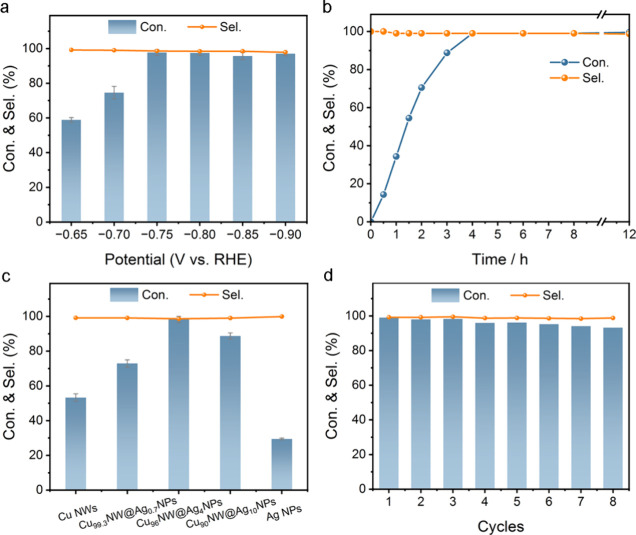
(a) Potential-dependent conversion (Con.) of EYA and selectivity (Sel.) for VYA over Cu_96_NW@Ag_4_NP hybrids studied over a 4 h reaction duration. (b) Time-dependent EYA Con. and VYA Sel. over Cu_96_NW@Ag_4_NP hybrids at −0.75 V. (c) EYA Con. and VYA Sel. for different catalysts at −0.75 V over a 4 h reaction duration. (d) Cycle-dependent EYA Con. and VYA Sel. over Cu_96_NW@Ag_4_NP hybrids at −0.75 V.

We first collected linear scanning voltammetry (LSV) curves across a potential range of 0 to −1.1 V for the Cu_96_NW@Ag_4_NP hybrids with and without the addition of EYA into the electrolyte (Figure S9). The presence of EYA led to a decrease in current density at potentials more negative than −0.6 V, attributed to its adsorption on the catalyst.^51^ Consequently, we focused on exploring the potential-dependent conversion of EYA and selectivity for VYA over Cu_96_NW@Ag_4_NP hybrids at potentials below −0.6 V during a 4 h reaction duration. As illustrated in Figure 2a, the conversion of EYA initially increased with the negative potential until reaching over 97% at −0.75 V, then maintained high levels at more negative potentials. Despite variations in potential, the selectivity consistently exceeded 98%, indicating independence from potential fluctuations. Additionally, the conversion of EYA and selectivity for VYA over Cu_96_NW@Ag_4_NP hybrids were evaluated as a function of reaction time at −0.75 V. The results, displayed in Figure 2b, indicate that a 4 h duration is sufficient for the complete conversion of EYA, translating to a reaction rate of approximately 141 μmol cm^–2^ h^–1^. Notably, the VYA selectivity remained high (>98%) throughout the reaction and did not decline even when the potential was sustained for 12 h, 8 h beyond the consumption of EYA. This suggests that the product alkenes do not readsorb over the Cu_96_NW@Ag_4_NP hybrids for further reduction.

In light of these preliminary results, we compared the performance of CuNW@AgNP hybrids at three Cu/Ag atomic ratios (i.e., 99.3/0.7, 96/4, 90/10) with monometallic Cu NWs and Ag NPs under identical conditions (i.e., −0.75 V, 4 h reaction). Consistent with previous studies that have highlighted the excellent selectivity of monometallic Cu- and Ag-based catalysts in the ESH of alkynes,^13,14,52^ all catalysts evaluated in this study demonstrated near-unity VYA selectivity (Figure 2c). However, the conversion rates for Cu NWs and Ag NPs were only 54% and 29%, respectively. To achieve a 99% conversion of EYA at −0.75 V, Cu NWs required up to 12 h, while Ag NPs managed only a 67% conversion under the same conditions (Figures S10 and S11). It is worth noting that the low conversion rate was not due to nonoptimization of the applied potential, as no obvious increase in EYA conversion was observed when varying the potential over Cu NWs and Ag NPs (Figures S12 and S13). Interestingly, the integration of relatively low-activity Ag NPs with Cu NWs remarkably enhanced the conversion rate of EYA over both individual components. As the Ag content increased, a volcano-shaped trend emerged, with the highest EYA conversion (99%) observed for Cu_96_NW@Ag_4_NP hybrids. This peak conversion coincided with the largest current density as indicated by the LSV curves (Figure S14a). Based on the current and the amount of VYA produced, we further calculated the VYA Faradaic efficiency and found that the Cu_96_NW@Ag_4_NP hybrids (14.6%) also exhibit a markedly higher VYA Faradaic efficiency compared to Cu NWs (8.1%) and Ag NPs (4.7%). Moreover, the Cu_96_NW@Ag_4_NP hybrids maintained over 90% conversion of EYA and a VYA selectivity of over 98% after 8 cycles (Figure 2d), and the morphology and the electronic properties of the hybrids after reaction did not change significantly (Figure S15), underscoring the high activity, selectivity, and durability of Cu_96_NW@Ag_4_NP hybrids for the ESH of EYA.

### Mechanistic Studies of ESH over CuNW@AgNP Hybrids

**Figure 3:**
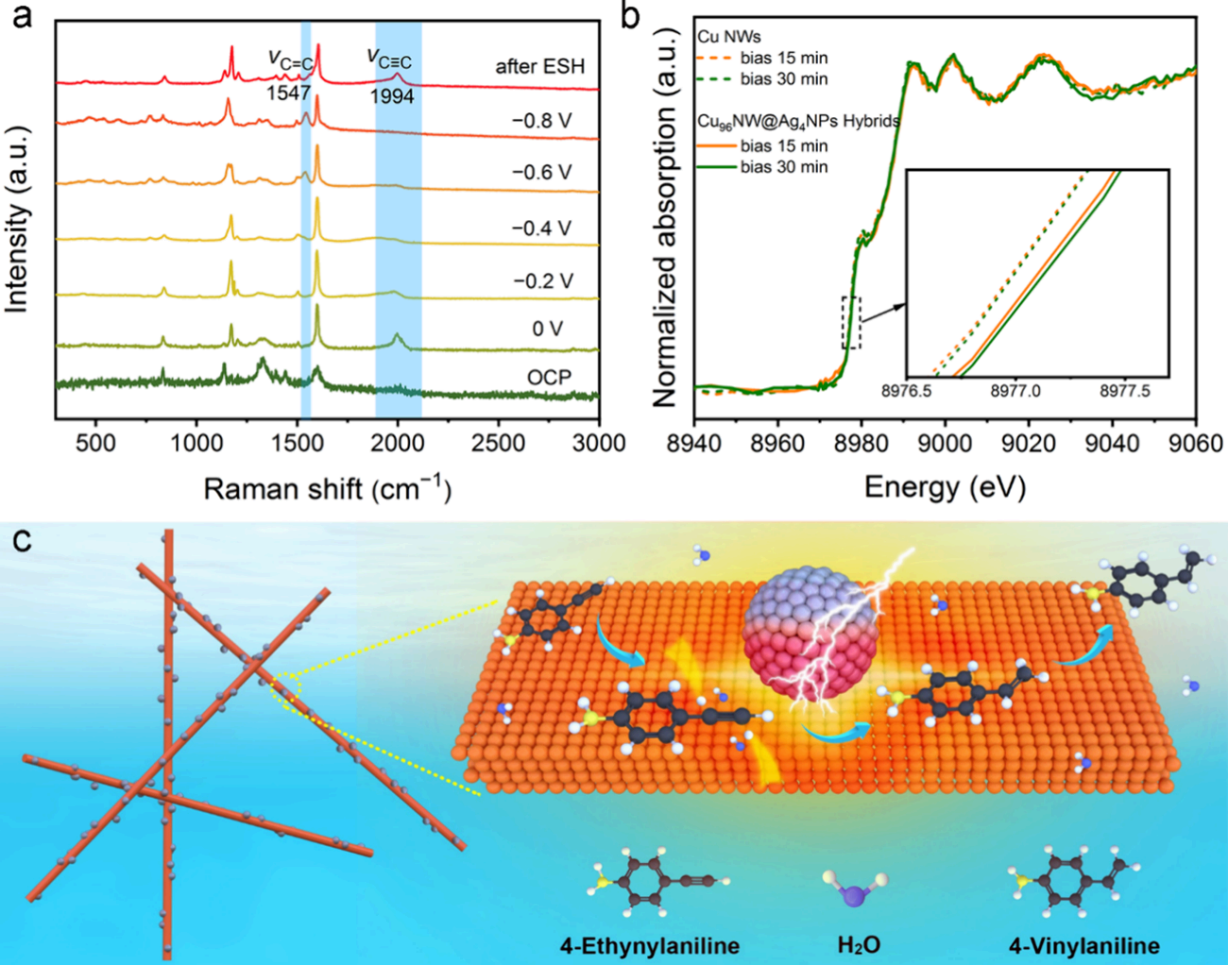
(a) Potential-dependent operando Raman spectra measured on Cu_96_NW@Ag_4_NP hybrids. (b) Cu K-edge XANES spectra measured over time on Cu NWs and Cu_96_NW@Ag_4_NP hybrids at −0.75 V vs RHE. (c) Schematic illustration of the ESH of EVA to VYA over CuNW@AgNP hybrids.

To gain insights into the ESH of EYA, we conducted operando Raman spectroscopy tests to monitor the transition of intermediate species over the Cu_96_NW@Ag_4_NP hybrids. As depicted in Figure 3a, in contrast to the open-circuit potential (OCP), the ν(C≡C) stretching vibration of EYA at 1994 cm^**–**1^ appeared when a potential of 0 V was applied, indicating the adsorption of EYA on the hybrids.^53,54^ Notably, the ν(C≡C) vibration peak is red-shifted compared to that of solid EYA (2090 cm^–1^, Figure S16), suggesting that EYA molecules preferentially adsorb in a horizontal configuration on the Cu surface through a σ–π-C_2_H_2_–Ar configuration.^53,55^ As the potential shifted negatively from 0 to −0.6 V, the ν(C≡C) vibration peak decreased gradually and completely disappeared at −0.8 V. Concomitantly, a new peak at 1547 cm^–1^, assigned to the symmetric C=C stretch modes of π-bonded ethylene, emerged at −0.4 V and intensified as the potential moved to −0.8 V, evidencing the formation of VYA on the hybrids. These results suggest that the hydrogenation was initiated at a potential earlier than −0.4 V, and the process accelerated at more negative potentials. With regard to Cu NWs, signals of EYA and VYA were also observed, confirming the occurrence of ESH of EYA to VYA, but the onset potential for the appearance of the VYA peak (i.e., −0.6 V) is more negative, and the peak intensity is way lower (Figure S17). Overall, these operando Raman results confirm faster ESH kinetics on Cu_96_NW@Ag_4_NP hybrids compared to Cu NWs, underlining the superior catalytic performance of the hybrid material.

Provided the modified electronic structure of Cu being crucial for the enhanced activity, it should maintain distinct between the hybrids and the Cu NWs during the ESH process. To testify this, we conducted operando XAS measurements to probe the valence state of Cu in both the Cu NWs and the hybrids. Interestingly, the chemical state of Cu in the hybrids is consistently more positive than that in the Cu NWs during the ESH (Figure 3b). This observation echoes the significant role of Ag in altering the electronic environment of Cu, thereby influencing its catalytic behavior.

To fundamentally understand the origin of the superior activity of the CuNW@AgNP hybrids, we implemented density functional theory (DFT) calculation to examine the ESH pathways over the hybrids in comparison with Cu NWs. The ESH process of EYA (C_8_H_7_N) to VYA (C_8_H_9_N) using H_2_O as a hydrogen source involves several steps^42,56,57^: (i) adsorption of EYA on the catalyst (C_8_H_7_N*); (ii) two consecutive hydrogen transfer steps involving H_2_O-derived H*, transforming C_8_H_7_N* to C_8_H_8_N* and then C_8_H_9_N*; (iii) desorption of C_8_H_9_N* from the catalyst to yield VYA (C_8_H_9_N) (Figure 3c). Additionally, if C_8_H_9_N* undergoes two further hydrogenation steps, the side product 4-ethylaniline (C_8_H_11_N*) would be formed. The optimized geometries of the Cu NWs and CuNW@AgNP hybrids are shown in Figure S18, with the molecules modeled to lay horizontally on the catalyst surface, reflecting insights gained from Raman spectroscopy results.

**Figure 4:**
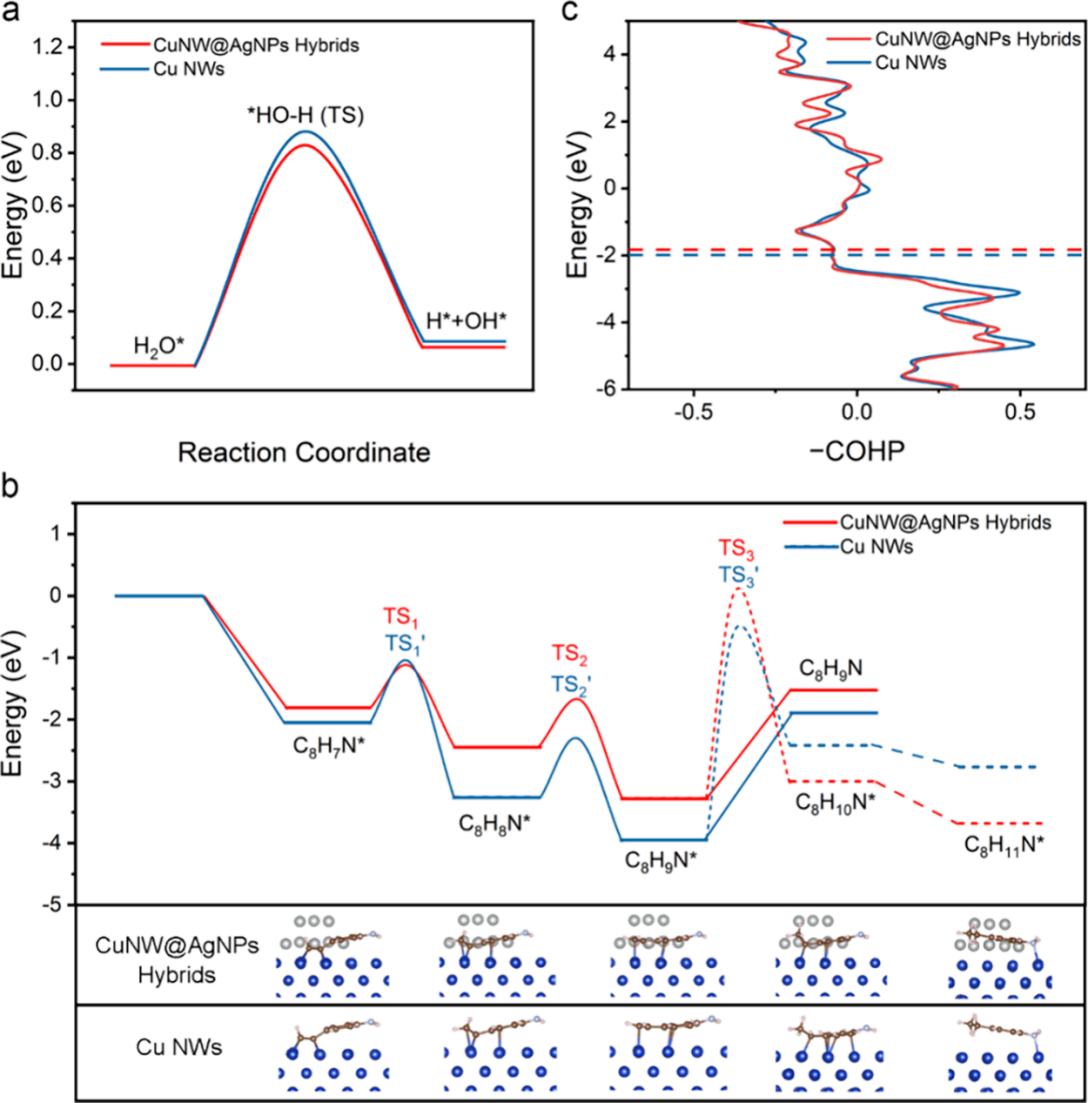
Free energy diagram for (a) water dissociation and (b) hydrogenation of EYA over Cu NWs and CuNW@AgNP Hybrids. (c) Crystal orbital Hamilton populations (COHP) of Cu–C atoms for Cu NWs and for CuNW@AgNP hybrids with C_8_N_9_N* adsorbed.

Given that H*, derived from H_2_O, participates in the reaction, we began by calculating the transition state energy barrier for the decomposition of H_2_O into H* on both Cu NWs and CuNW@AgNPs (Figure 4a and Figures S19 and S20). The similar energy barrier values, coupled with comparable hydrogen-evolution-reaction (HER) current densities observed in the LSV curves of both Cu NWs and CuNW@AgNP hybrids (Figure S14b), indicate that the decomposition of H_2_O is not the distinguishing factor for the enhanced activity observed with the hybrids.

We then explored the thermodynamic behavior of the ESH of EYA to VYA by calculating the Gibbs free energies (Δ*G*) and transition state energy barriers of key intermediates on both Cu NWs and CuNW@AgNP hybrids (Figure 4b). In the two hydrogenation steps (specifically, C_8_H_7_N* + H* → C_8_H_8_N*, followed by C_8_H_8_N* + H* → C_8_H_9_N*), the corresponding transition state energy barriers for the hybrids (TS_1_: 0.660 eV and TS_2_: 0.570 eV) are lower than those for Cu NWs (TS_1_’: 1.000 eV and TS_2_’: 0.936 eV), indicating easier hydrogenation on the hybrids. In the desorption step (C_8_H_9_N*→ C_8_H_9_N), benefiting from weakened alkene adsorption, the hybrids also display a lower desorption barrier compared to Cu NWs (cf. 1.757 eV vs 2.050 eV), suggesting a more facile desorption of the alkene from the hybrids. Noteworthily, for both catalysts, the alkene desorption barrier is higher than the transition state energy barriers of the two preceding hydrogenation steps, indicating that the desorption step is the rate-determining step that determines the overall activity of the ESH. Therefore, we can conclude that both hydrogenation and desorption occur more readily on the hybrids than on the pristine Cu NWs. Particularly, the more facile desorption of alkenes explains the superior conversion rate of the hybrids. An additional interesting finding is that all intermediates produced in ESH process are less strongly bound on the hybrids compared to the Cu NWs—as predicted by the scaling relations,^58^ highlighting the unique role of Ag in modulating the electronic properties of Cu. Moreover, the transition state energy barriers for further hydrogenation of alkenes (C_8_H_9_N*) to C_8_H_10_N* (TS_3_: 3.029 eV and TS_3_’: 3.686 eV) on both the hybrids and Cu NWs are significantly higher than the corresponding desorption barriers. This higher barrier for further hydrogenation accounts for the high selectivities toward alkene products rather than alkane products during the hydrogenation of EYA.

So far, we have provided rationale for the enhanced activity and high selectivity of the CuNW@AgNP hybrids, but the mechanism by which the integration of Ag NPs effectively weakens the adsorption of intermediates remains unclear. To address this, we further analyzed the crystal orbital Hamilton populations (COHP) of Cu–C atoms for Cu NWs and for CuNW@AgNP hybrids with adsorbed C_8_N_9_N* (Figure 4c). The analysis showed that the antibonding states in CuNW@AgNP hybrids are significantly more populated than in Cu NWs, leading to reduced adsorption strength of C_8_N_9_N*. Additionally, the integrated-crystal orbital Hamilton population (ICOHP) serves as another quantitative measure of bonding strength. Generally, a more positive ICOHP value indicates weaker binding.^59^ The ICOHP value for CuNW@AgNP hybrids is more positive than that for Cu NWs (cf. −1.83 eV vs −1.99 eV), further supporting the observation of easier desorption of C_8_N_9_N* from the hybrids. This computational evidence helps clarify why the presence of Ag in the Cu NWs modifies the electronic structure in a way that weakens adsorption, enhancing the overall catalytic performance.

In the current computations, the varying degrees of charge deficiency in Cu, as experienced by the CuNW@AgNP hybrids with different Ag contents, were not factored in. Nevertheless, the volcano plot of the conversion rate as a function of Ag content—with the Cu_96_NW@Ag_4_NP hybrids at the apex—conforms to the Sabatier principle. This alignment suggests that the Cu_96_NW@Ag_4_NP hybrids featuring moderately decharged Cu represent the optimal catalyst, providing the right binding strength for adsorbates. In contrast, the Cu in Cu_99.3_NW@Ag_0.7_NPs and Cu_90_NW@Ag_10_NPs are insufficiently and overly decharged, respectively. This disparity unidirectionally impacts the binding behavior of a series of intermediate species in the ESH process, preventing balanced binding between these intermediates and ultimately hindering optimal activity.

### Universality of ESH of Alkynes over CuNW@AgNP Hybrids

To investigate the generalizability of CuNW@AgNP Hybrids for electrocatalytic alkyne semihydrogenation reactions, a variety of alkynes with varied substituent groups were tested as substrates. As detailed in Table 1, most of these alkynes were converted to their corresponding alkenes with remarkable selectivity (97%–99%) and high conversion yields (91%–99%). The selectivity and conversion for arylalkynes were excellent regardless of the substituent position (ortho-, meta-, or para-position, Entries 1–3) or the presence of an amino group (Entry 4). However, the electronic character of the substituent at the para-position proved significant. For example, the electron-donating methyl group (−CH_3_) resulted in a lower conversion of only 70% (Entry 5), while the electron-withdrawing group (−Cl,) achieved a conversion of 97% (Entry 6). The thienyl group, typically challenging for metal catalysts, had nearly no impact on the activity and selectivity of the product, highlighting the robust nature of the CuNW@AgNP hybrids (Entry 7).

**Table 1:**
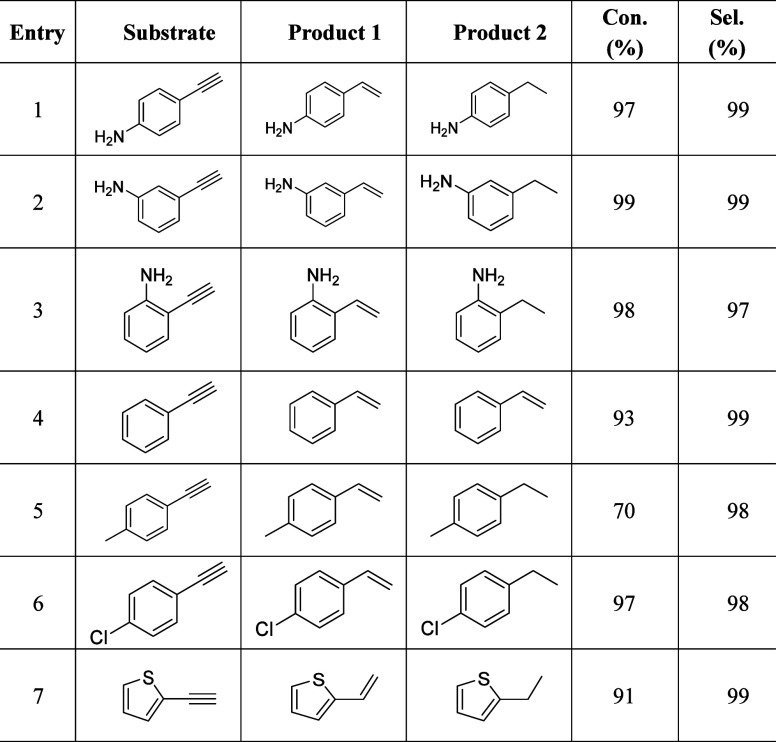
Substrate Scope for ESH of Alkynes Using Cu_96_NW@Ag_4_NP Hybrids as Catalysts at −0.75 V for 4 h

Finally, we would like to add a further discussion regarding the fabrication of the CuNW@AgNP hybrids. We also created the hybrids with Cu–Ag bimetallic interfaces via a chemical method (Figure S21), namely, a galvanic replacement reaction between Cu NWs and Ag ion precursors, and tested them for the ESH of EYA to VYA (Figure S22). The performance results were very similar to those obtained with the welding-resultant hybrids, further underscoring the importance of forming bimetallic interfaces for activity enhancement. However, in this work, we favored the welding-based method over the chemical approach, as the latter, at the expense of etching the Cu NWs, could inevitably induce surface structural changes to the Cu NWs, complicating the elucidation of the origin of any activity differences. Additionally, the welding of presynthesized, well-defined separate entities allows for easy tuning of the size and uniformity of Ag NPs, as well as the number density of Ag NPs attached onto the Cu NWs.

## Conclusions

In summary, we have utilized a welding method to create rationally designed Cu–Ag hybrids (CuNW@AgNPs) with Ag NPs controllably fused to Cu NWs, which serve as efficient electrocatalysts for the ESH of alkynes to alkenes. Through a comprehensive series of characterizations, we have demonstrated that the electron transfer from Cu to Ag within the CuNW@AgNP hybrids leads to a significant enhancement in alkyne conversion rates—approximately 2 to 4 times higher compared to isolated Ag NPs or Cu NWs—while maintaining over 99% selectivity for alkene products. Combining operando and computational investigations, we confirmed that the electron-depleted Cu sites, created by this electron transfer, significantly weaken the adsorption of alkenes. This weakening facilitates the potential-limiting step of alkene desorption, thereby effectively boosting ESH activity. Moreover, the thermodynamically unfavorable conditions for overhydrogenation of alkenes to alkanes over the hybrids ensure intrinsically high alkene selectivity. This work presents a novel approach to enhancing the selective ESH of alkynes through intentionally engineered hybrid electrocatalysts that specifically target the rate-determining step.

## Experimental Section

### Chemicals

Copper(II) chloride dihydrate (CuCl_2_·2H_2_O, 99.999%), silver nitrate (AgNO_3_, 99.8%), d-(+)-glucose (>99.5%), hexadecylamine (>98%), oleylamine (90%), and anhydrous ethanol were all purchased from Aladdin. Hexane (99.9%) was obtained from Kelong. All chemicals were used as received without further purification. Deionized water (18.2 MΩ·cm) used for preparing solutions was produced by an ultrapure water purification system (Milli-Q Advantage A10).

### Preparation of Cu NWs

In a typical synthesis of the Cu NWs,^47^ 22 mg of CuCl_2_·2H_2_O, 50 mg of D-(+)-glucose, and 180 mg of hexadecylamine were dissolved in 10 mL of deionized water and stirred overnight at room temperature. Subsequently, the solution was transferred to an oil bath where the temperature was raised to 100 °C and maintained for 8 h. After the reaction, the solution was cooled to room temperature, and the Cu NWs were washed five times with an ethanol/hexane mixture (1:1 volume ratio). The Cu NWs were then collected by centrifugation at 9500 rpm for 5 min and redispersed in hexane.

### Preparation of Ag NPs

In a typical synthesis of the Ag NPs,^60^ 1 mmol of AgNO_3_ was mixed with 20 mL of oleylamine at room temperature and then heated to 60 °C. The temperature was maintained until the granular AgNO_3_ crystals had completely dissolved. Subsequently, the temperature of the solution was rapidly increased to 210 °C and maintained for 1 h. A nitrogen atmosphere and magnetic stirring (∼700 rpm) were maintained throughout the entire synthesis process. After cooling the reaction system to room temperature, the dark brown organosol obtained was washed with acetone and redispersed in hexane.

### Preparation of CuNW@AgNP hybrids

The as-prepared Cu NWs were evenly dispersed in hexane by sonication. The Ag NPs obtained earlier were dispersed in 10 mL of hexane to form a colloidal Ag NP sol (atomic concentration: 0.052 M). Subsequently, 125, 50, and 10 μL of the Ag NP sol were added to the Cu NWs solution and sonicated for 5 min. After centrifugation (6000 rpm) and drying in a vacuum oven at room temperature for 4 h, the Cu_90_NW@Ag_10_NPs, Cu_96_NW@Ag_4_NPs, and Cu_99.3_NW@Ag_0.7_NP hybrids were obtained, respectively.

### Characterizations

Transmission electron microscopy (TEM), high-resolution TEM (HRTEM), energy dispersive X-ray (EDX) elemental maps, and high-angle-annular-dark-field scanning TEM (HAADF-STEM) were conducted using a Talos 200 S/TEM (Thermo Fisher Scientific) operated at 200 kV. X-ray photoelectron spectroscopy (XPS) measurements were performed on an ESCALAB 250Xi with a monochromatic Al Kα X-ray source under ultrahigh vacuum conditions. All peaks were calibrated using the C 1s spectrum at a binding energy of 284.8 eV. X-ray diffraction (XRD) patterns were recorded using a PANalytical X’Pert Powder Advance instrument with Cu Kα radiation. Inductively coupled plasma-optical emission spectrometry (ICP-OES) was carried out on a Spectro GREEN model. Quantitative analysis of the liquid products was conducted using a gas chromatograph (GC, Agilent 8860) equipped with a thermal conductivity flame ionization detector (FID) and a WAX capillary column (0.25 mm in diameter, 30 m in length). The injection temperature was set at 260 °C, and nitrogen was used as the carrier gas at a flow rate of 1.5 mL/min. X-ray absorption fine structure (XAFS) spectroscopy was performed using a *RapidXAFS* 2 M (Anhui Absorption Spectroscopy Analysis Instrument Co., Ltd.) in transmission mode at 20 kV and 20 mA, and the Si (553) spherically bent crystal analyzer with a radius of curvature of 500 mm for Cu. The XAS spectra were analyzed with the ATHENA software package. Cu NWs and CuNW@AgNP hybrid powders were loaded onto carbon paper for in situ Raman and in situ XANES tests.

### In Situ Raman Spectroscopy

The electrochemical in situ Raman measurements were conducted using a HORIBA HR Evolution spectrometer in a commercial electrolytic cell equipped with a quartz window. A 633 nm He–Ne laser served as the excitation source. A mixed solution containing 10 mL of 1.0 M KOH and 4 mL of dioxane with 0.4 mmol EYA was added to the electrolytic cell. Raman spectra were collected from the same point, ranging from open-circuit potential (OCP) to −0.8 V, using an Olympus 50× objective. Each spectrum had a collection time of 30 s.

### Electrochemical Measurements

The catalyst ink was prepared by ultrasonically dispersing 1 mg of sample powder in 500 μL of ethanol for 30 min. Subsequently, 5 μL of nafion solution (5% by weight) was added to the ink and sonicated for an additional 5 min. The homogeneous catalyst ink was then evenly applied to carbon paper (loading area: 1.0 cm^2^) to serve as the working electrode. Electrochemical measurements were conducted in a divided two-compartment electrochemical cell, which included a working electrode, a Pt gauze counter-electrode, and a Hg/HgO reference electrode, using a CHI760e electrochemical workstation (Shanghai Chenhua). All potentials were referenced to the reversible hydrogen electrode (RHE) according to the following equation:
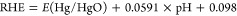
1

A 14 mL electrolyte solution, comprising a 1.0 M KOH solution (with 4.0 mL dioxane and 10.0 mL H_2_O, pH = 13.6), was added to both the cathodic and anodic compartments, separated by a Nafion-N117 membrane (Sigma-Aldrich). In the cathodic compartment, 0.4 mmol of alkynes was added and stirred to form a homogeneous solution. Chronoamperometry was then conducted at constant biases ranging from −0.65 to −0.90 V vs RHE for 4 h. Subsequently, products from the cathodic compartment were extracted with dichloromethane (DCM). The DCM phase was analyzed by gas chromatography (GC) to determine the conversion yields. These yields were calculated based on a standard calibration curve, using dodecane as an internal standard. Linear sweep voltammetry (LSV) curves were recorded within a potential range of 0 to −1.0 V vs RHE at a scan rate of 10 mV s^–1^. The alkyne conversion (Con.) and alkene selectivity (Sel.) were then calculated as follows:
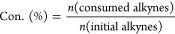
2
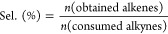
3

### Calculation of Faradaic Efficiency

The Faradaic efficiency for VYA was calculated using the following equations. Q(VYA) represents the amount of charges used to produce VYA, which is determined by multiplying the amount of VYA produced by Faraday constant and 2 (since the production of 1 mol of VYA consumes 2 mol of electrons). Q(total) is the total charge passing through the circuit, calculated by integrating the current–time (i-t) curves at a fixed potential.
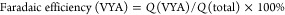
4
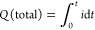
5

### Computational Details

All theoretical calculations (density functional theory (DFT) calculations) were performed using the Vienna ab initio simulation package (VASP, version 5.4.4). The electron–ion interaction was described using the projected augmented wave (PAW) potential. The electron exchange and correlation energies were estimated using the generalized gradient approximation (GGA) of the Perdew–Burke–Ernzerhof (PBE) functional. The Brillouin zone was sampled using a 2 × 2 × 1 Monkhorst–Pack grid. Settings included a 520 eV plane-wave energy cutoff, an energy convergence threshold of 10^–5^ eV, and a maximum residual force below −0.02 eV/Å. In this study, our slab model comprises an Ag(111) cluster added to the Cu(100) surface, which was utilized to calculate the free energy, adsorption energy, and desorption energy. The adsorption energy of phenylacetylene (*E*_ads_) was calculated using the following equation:
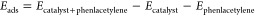
6where *E*_catalyst+phenylacetylene_, *E*_catalyst_, and *E*_phenylacetylene_ are the energies of the catalyst with absorbed phenylacetylene, catalyst, and phenylacetylene, respectively.

The Gibbs free energy change (Δ*G*) for each step of the electrocatalytic hydrogenation and the transition state (TS) for the overhydrogenation of styrene were calculated as follows:
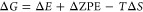
7where Δ*E* represents the energy difference between reactants and products for each reaction step, ΔZPE is the change in zero-point energy, and Δ*S* is the change in entropy. The zero-point energy of the adsorbates was determined from vibrational frequency calculations.

## Supporting Information Available

Figures S1–S22, including additional information regarding structural and electronic characterizations (TEM, HAADF-STEM, XRD, XPS) of samples, standard GC curves used for product quantification, electrochemical measurements, in situ Raman spectra, theoretical calculation models, and CuNW@AgNP hybrids prepared by a galvanic replacement reaction method (PDF)
