# Supplementary material for: Enhancing Electrocatalytic Semihydrogenation of Alkynes via Weakening Alkene Adsorption over Electron-Depleted Cu Nanowires
Source: ACS Nanosci Au. 2024 Aug 8;4(5):349–59. doi: 10.1021/acsnanoscienceau.4c00030 (PMC11487759; doi:10.1021/acsnanoscienceau.4c00030)
Supplement: Supplementary file 1 — ng4c00030_si_001.pdf [file ng4c00030_si_001.pdf]

SUPPORTING INFORMATION

Enhancing Electrocatalytic Semihydrogenation of Alkynes via Weakening Alkene Adsorption over Electron-depleted Cu Nanowires

Dan Luo,[a,+] Zhiheng Xie,[b,+] Shuangqun Chen,[a] Tianyi Yang,[a] Yalin Guo,[*,a] Ying Liu,[a] Zhouhao Zhu,[b] Liyong Gan[*,b] Lingmei Liu,[a] and Jianfeng Huang[*,a]

[a] State Key Laboratory of Coal Mine Disaster Dynamics and Control, Institute of Advanced Interdisciplinary Studies, School of Chemistry and Chemical Engineering, Chongqing University, Chongqing 400044, China

[b] College of Physics and Center of Quantum Materials and Devices, Chongqing University, Chongqing 401331, China

[+] These authors contributed equally to this work.

E-mail: guoyalin2022@cqu.edu.cn, ganly@cqu.edu.cn, jianfeng.huang@cqu.edu.cn

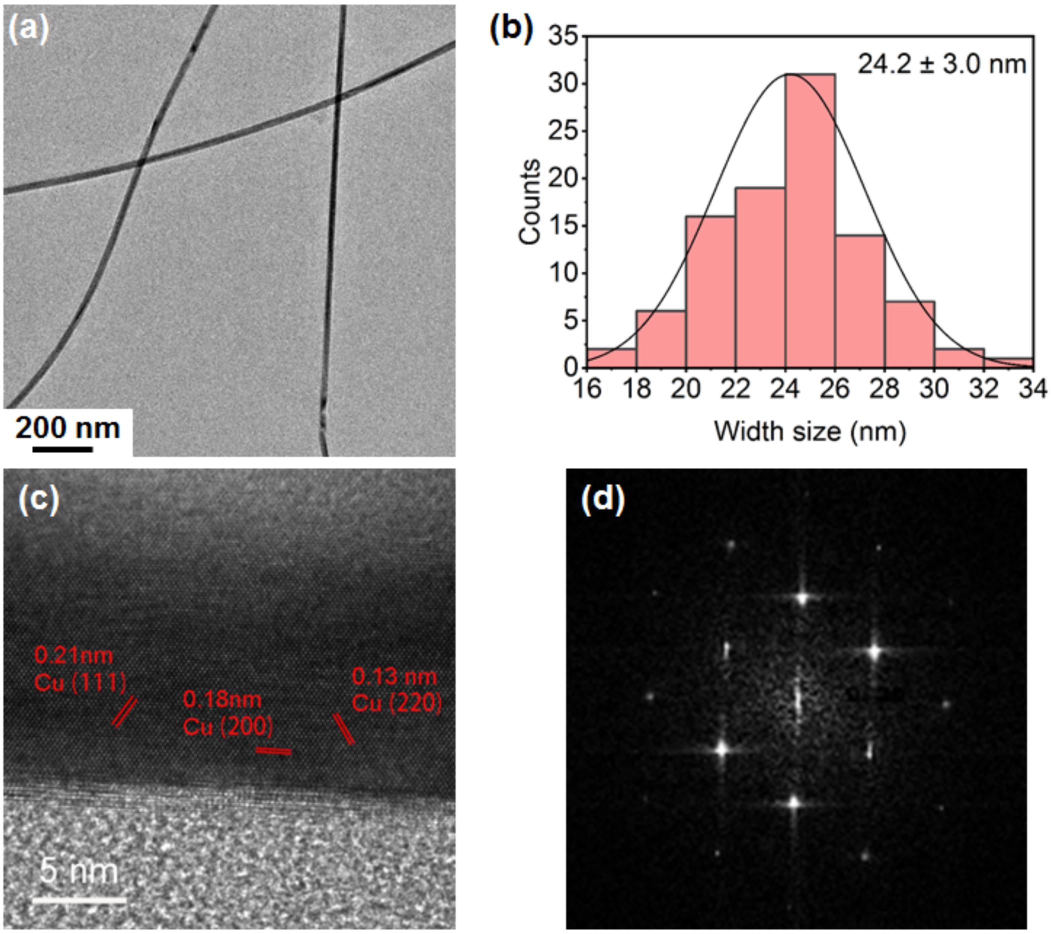

Figure S1. (a) TEM image and (b) width distribution of the Cu NWs with an average diameter of 24.2 ± 3.0 nm; (c, d) HRTEM and the corresponding FFT of one Cu NW.

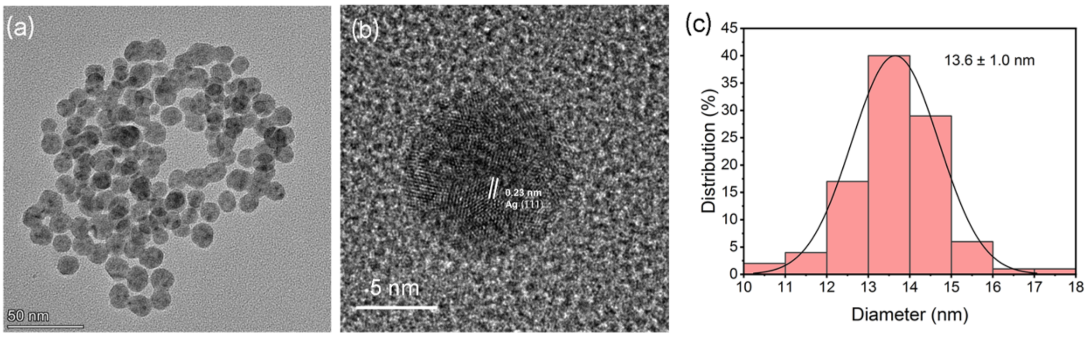

Figure S2. (a) TEM image, (b) HRTEM, and (c) size distribution of the Ag NPs with an average diameter of 13.6 nm.

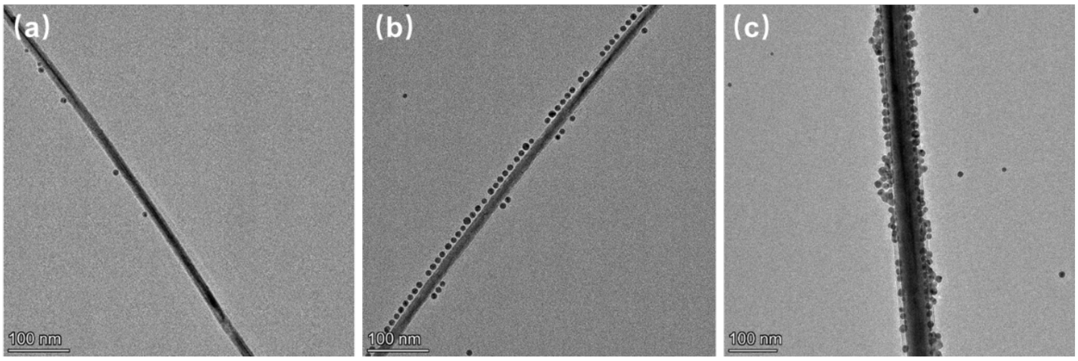

Figure S3. TEM images of (a) $Cu_{99.3}NW@Ag_{0.7}NPs$, (b) $Cu_{96}NW@Ag_{4}NPs$ and (c) $Cu_{90}NW@Ag_{10}NPs$ hybrids.

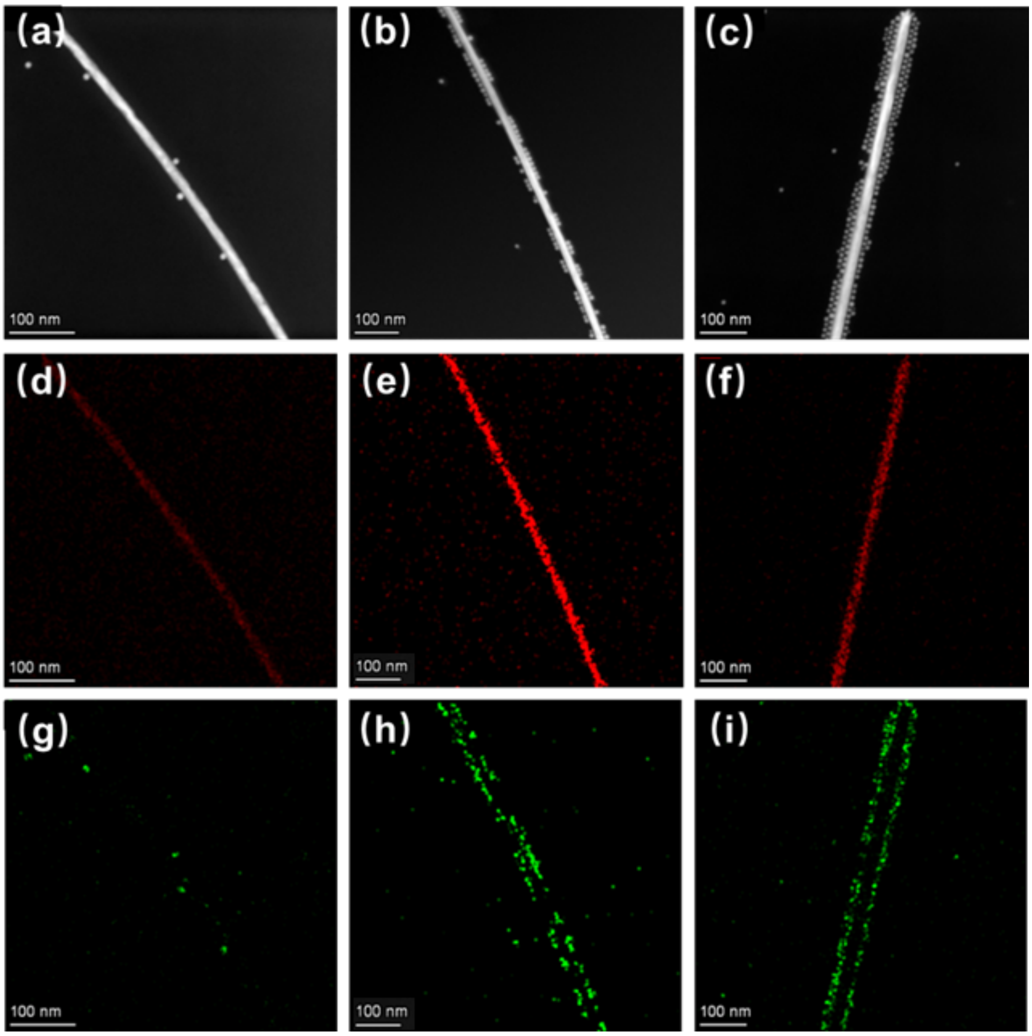

Figure S4. HAADF-STEM images and the corresponding EDX elemental mappings of Cu (red) and Ag (green) of the CuNW@AgNPs hybrids with three different Cu/Ag ratios: (a) $Cu_{99.3}$NW@$Ag_{0.7}$NPs, (b) Cu_{96}NW@Ag_{4}NPs and (c) Cu_{90}NW@Ag_{10}NPs hybrids.

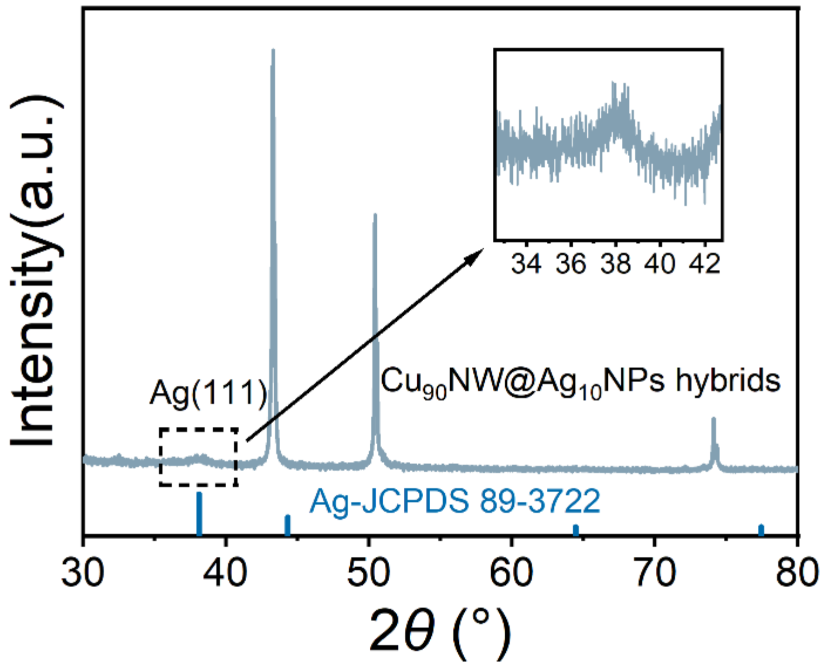

Figure S5. XRD pattern of $Cu_{90}NW@Ag_{10}NPs$ hybrids, with the boxed region enlarged in the inset, showing the diffraction peak from the Ag(111) planes.

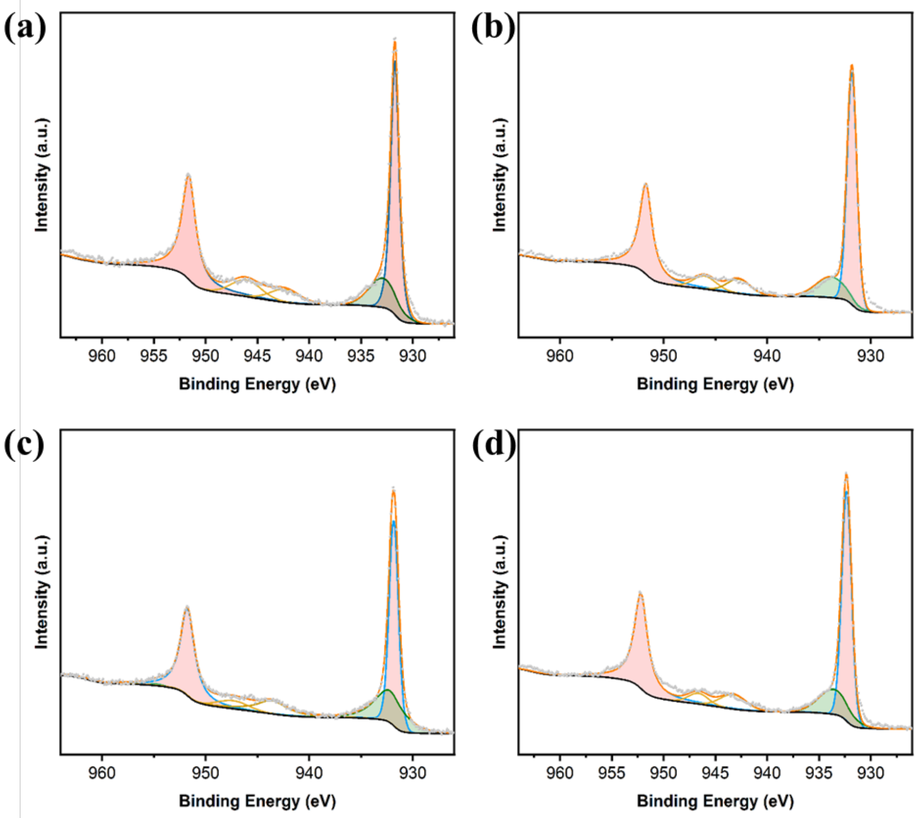

Figure S6. XPS Cu 2p spectra of (a) Cu NWs, (b) $Cu_{99.3}$NW@$Ag_{0.7}$NPs, (c) Cu_{96}NW@Ag_{4}NPs, and (d) Cu_{90}NW@Ag_{10}NPs hybrids.

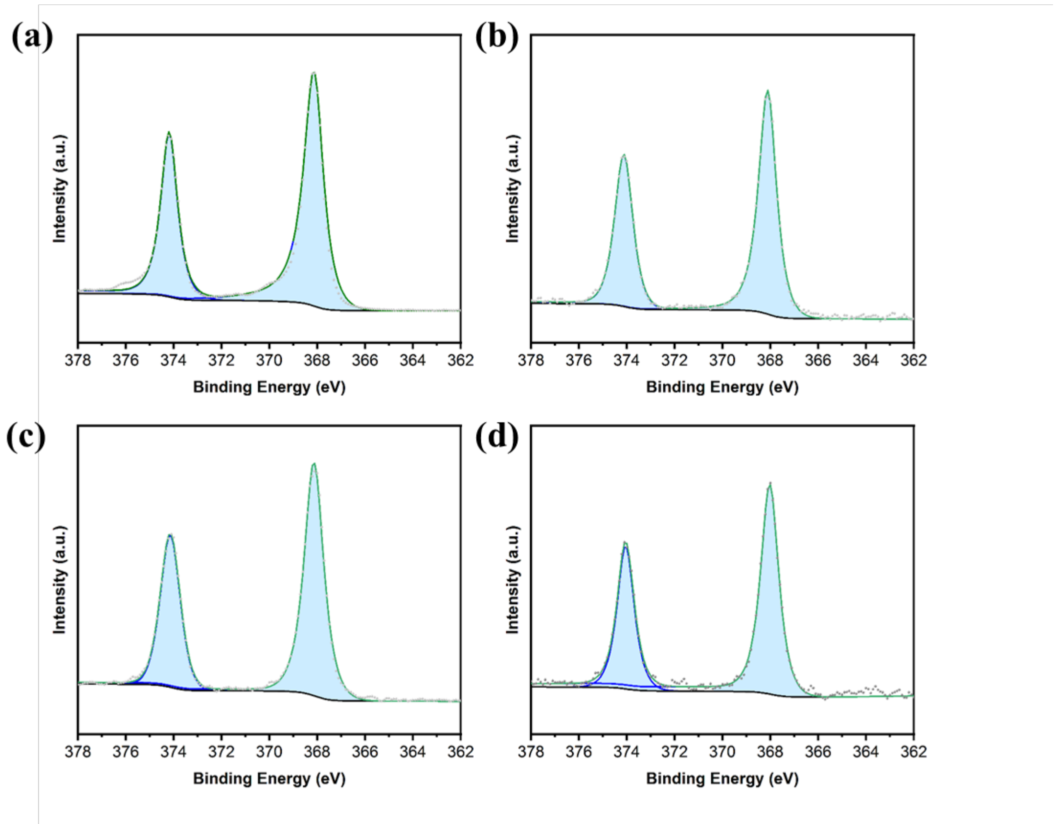

Figure S7. XPS Ag 3d spectra of (a) Ag NPs, (b) $Cu_{99.3}NW@Ag_{0.7}NPs$, (c) $Cu_{96}NW@Ag_4NPs$, and (d) $Cu_{90}NW@Ag_{10}NPs$ hybrids.

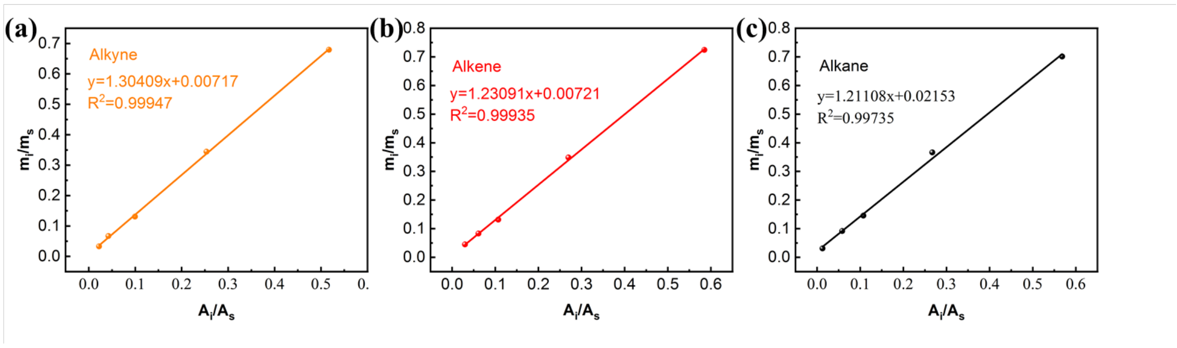

Figure S8. Standard curves used to quantify (a) alkynes (EYA), (b) alkenes (VYA), and (c) alkanes (4-ethylaniline).

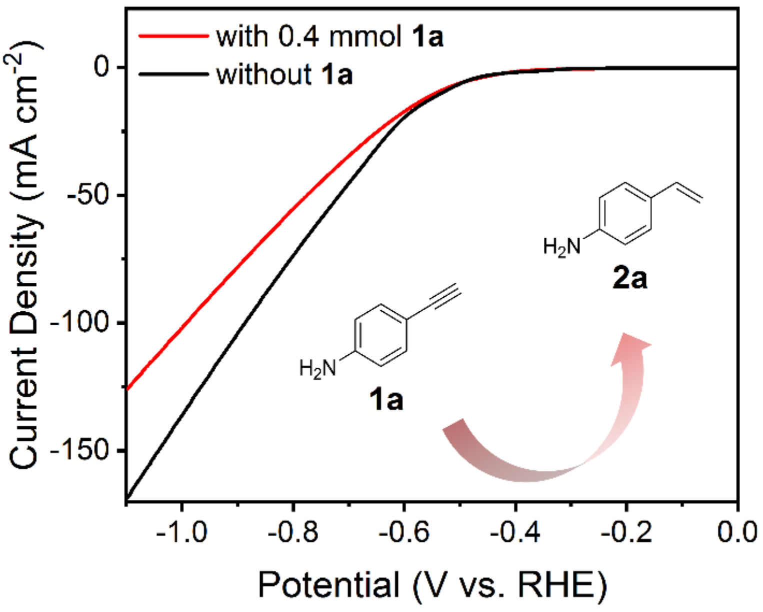

Figure S9. LSV curves of $Cu_{96}NW@Ag_4NPs$ in 1.0 M KOH solution [dioxane/H_2O, 2/5 (v/v)] at a scan rate of 5 mV s^{-1} with and without 0.4 mmol of EYA.

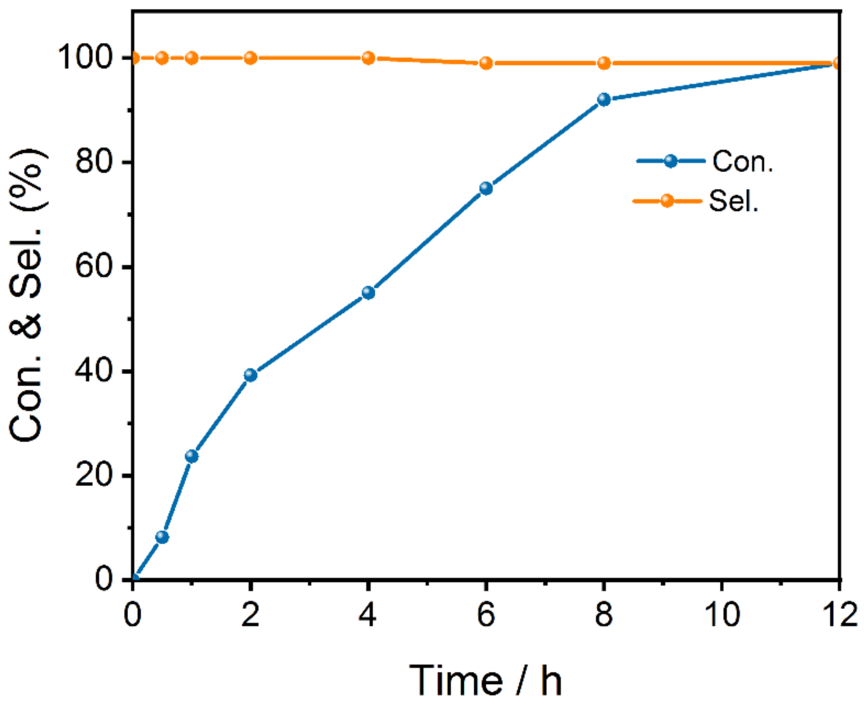

Figure S10. Time-dependent conversions (Con.) of EYA and selectivity (Sel.) for VYA over Cu NWs at −0.75 V.

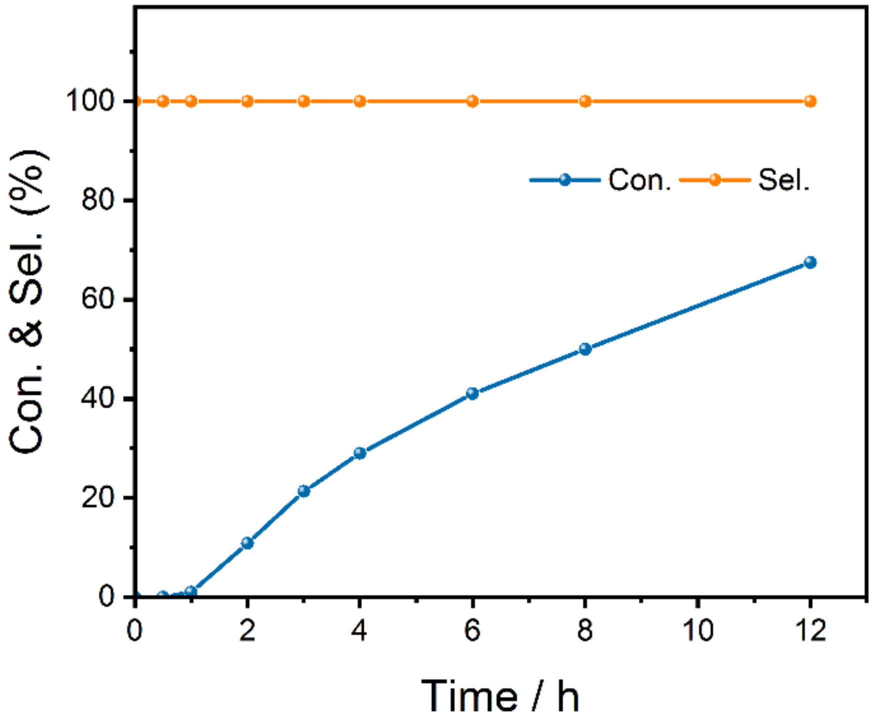

Figure S11. Time-dependent conversions (Con.) of EYA and selectivity (Sel.) for VYA over Ag NPs at −0.75 V.

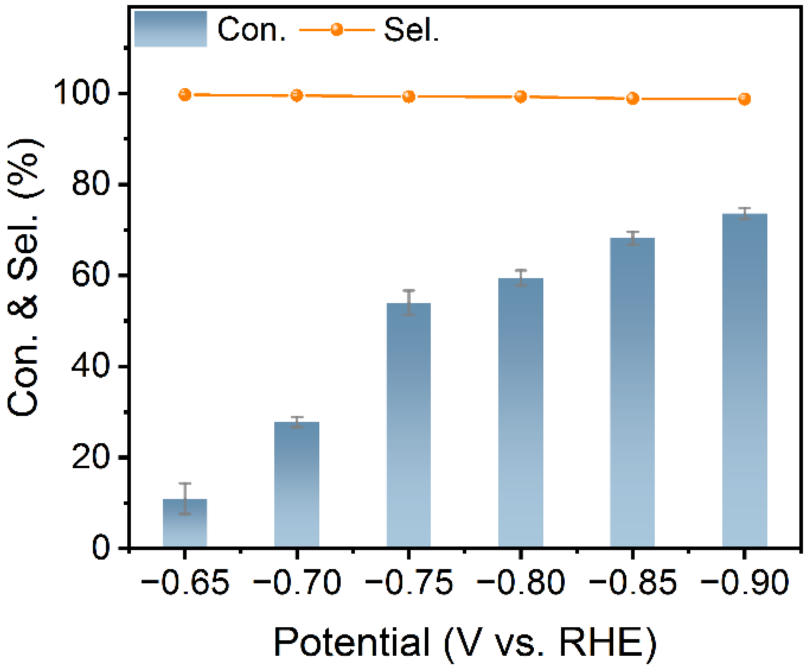

Figure S12. Potential-dependent conversions (Con.) of EYA and selectivity (Sel.) for VYA over Cu NWs within 4 h.

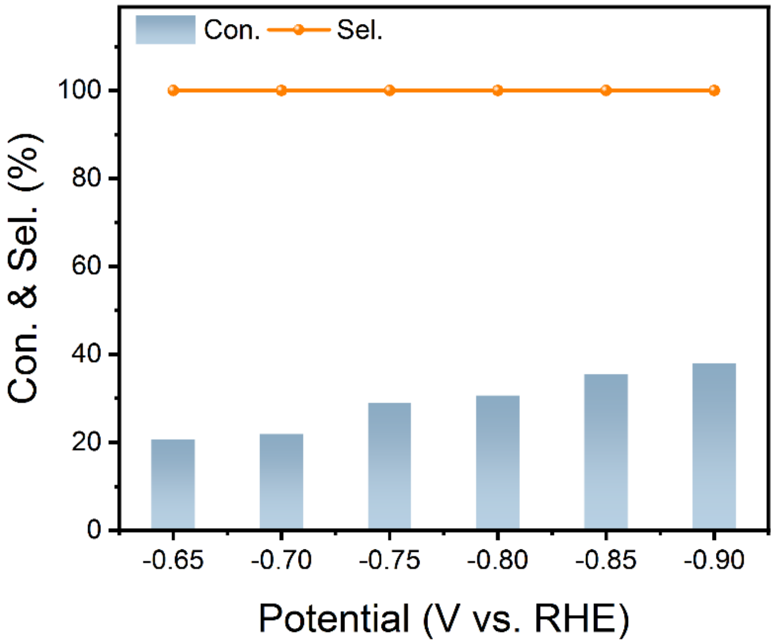

Figure S13. Potential-dependent conversions (Con.) of EYA and selectivity (Sel.) for VYA over Ag NPs within 4 h.

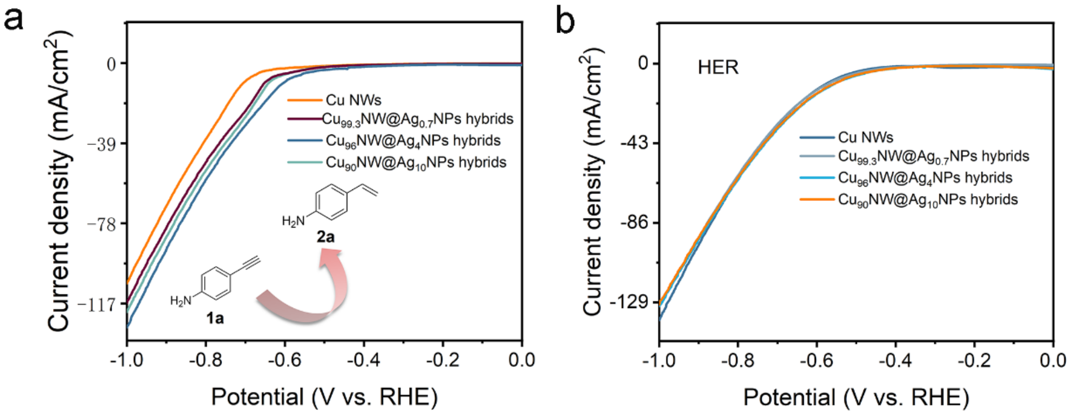

Figure S14. LSV curves of Cu NWs, $Cu_{99.3}NW@Ag_{0.7}NPs$, $Cu_{96}NW@Ag_{4}NPs$, and $Cu_{90}NW@Ag_{10}NPs$ in 1.0 M KOH solution [dioxane/H_2O, 2/5 (v/v)] at a scan rate of 5 mV s^{-1} (a) with and (b) without 0.4 mmol of EYA.

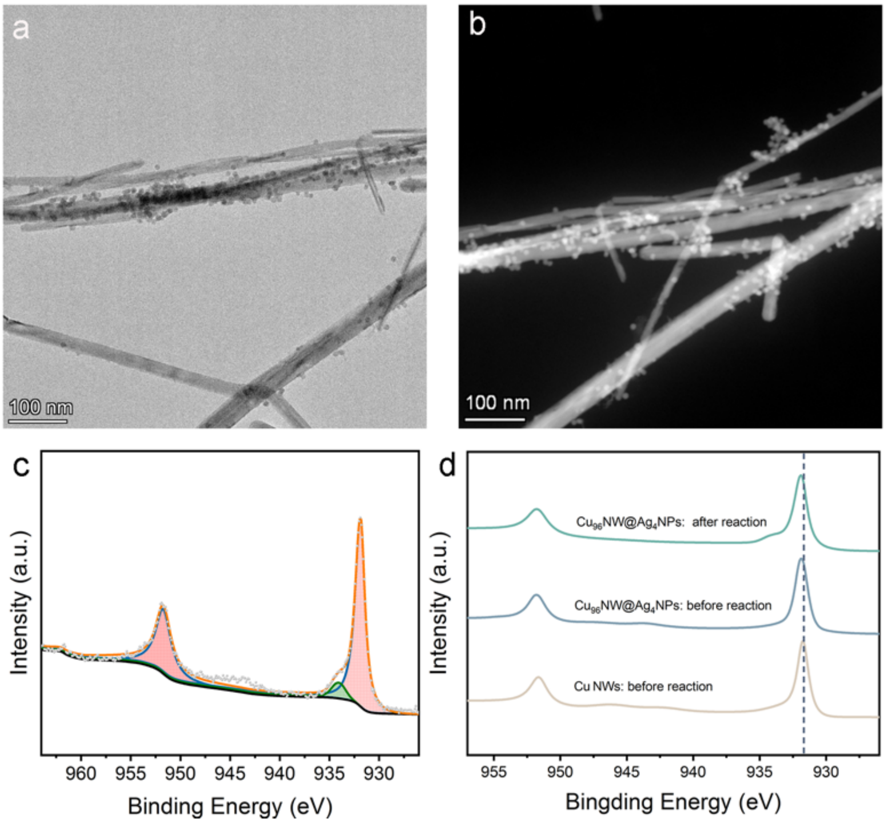

Figure S15. (a) TEM and (b) HAADF-STEM image (c) XPS Cu 2p spectra of Cu_{96}NW@Ag_4NPs hybrids after 4h's electrolysis at −0.75 V *vs.* RHE, (d) comparison between XPS Cu 2p spectra of Cu_{96}NW@Ag_4NPs hybrids before and after reaction and XPS Cu 2p spectra of Cu NWs.

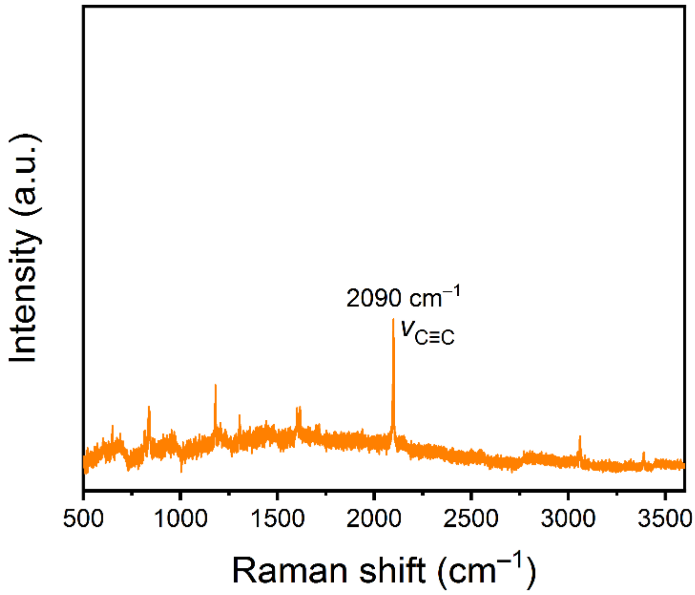

Figure S16. Raman spectra of 4-Ethynylaniline (EYA) powders.

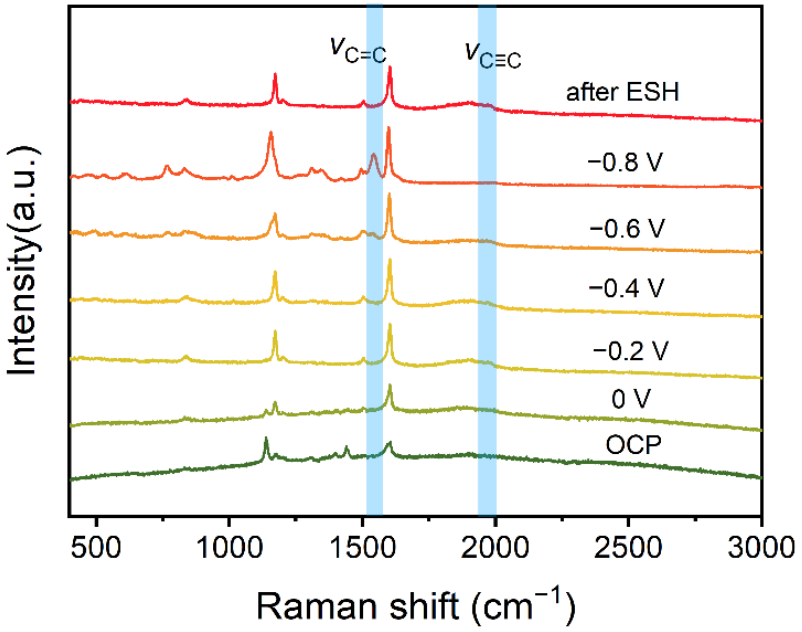

Figure S17. In situ potential-dependent Raman spectra measured on Cu NWs.

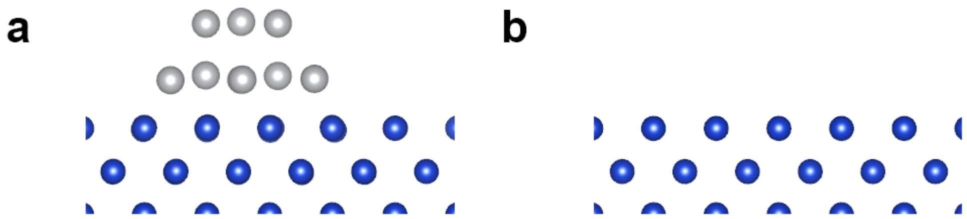

Figure S18. Theoretical calculation models of catalysts: (a) CuNW@AgNPs hybrids and (b) Cu NWs. Cu and Ag atoms are shown as blue and white spheres, respectively.

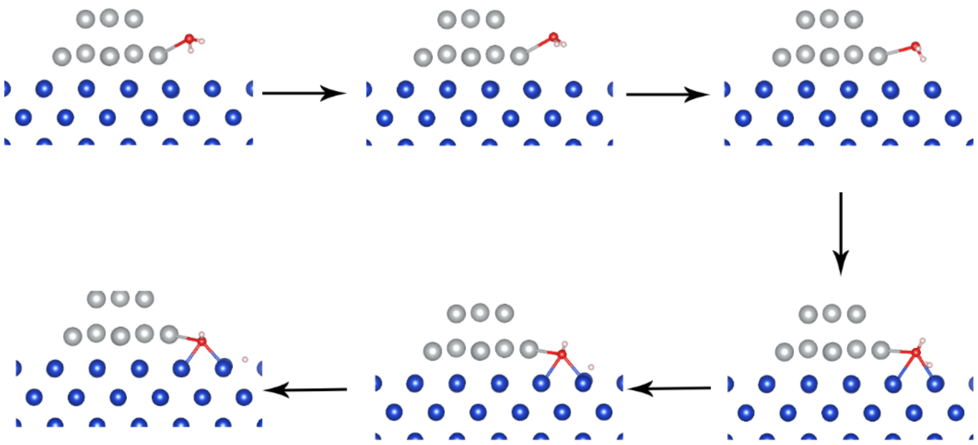

Figure S19. Transformation of adsorption configurations in H_2O decomposition on CuNW@AgNPs hybrids surface.

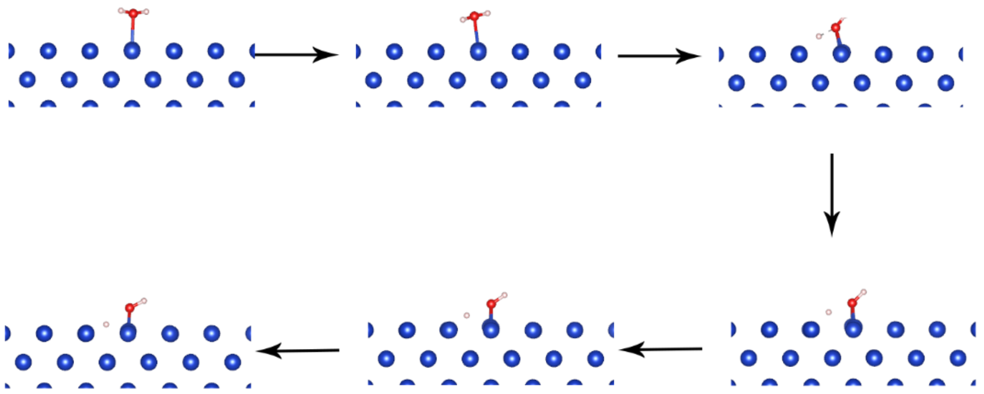

Figure S20. Transformation of adsorption configurations in H_2O decomposition on Cu NWs surface.

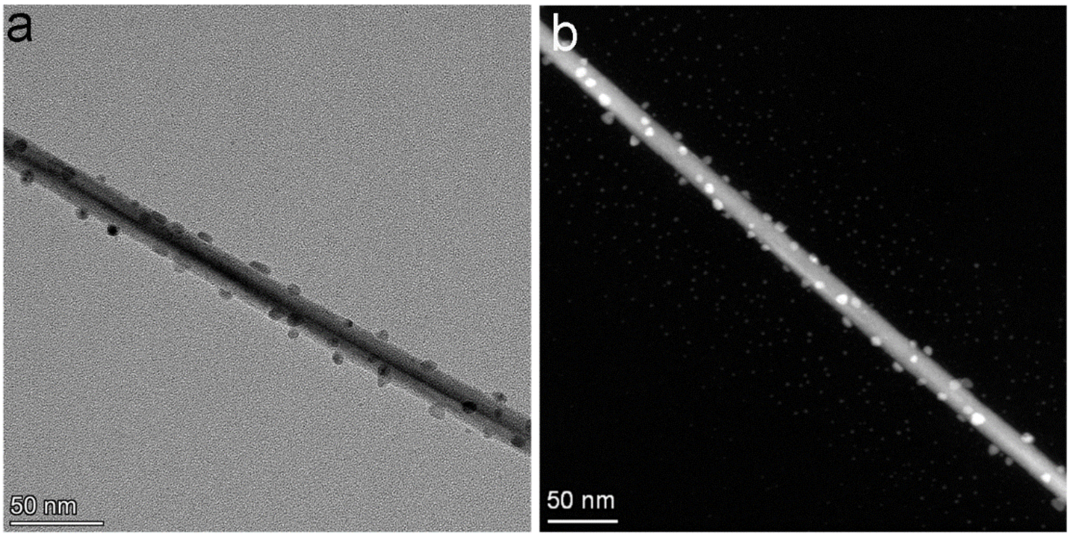

Figure S21. (a) TEM image and (b) HAADF-STEM image of CuNW@AgNPs-GRR bimetallic catalysts fabricated by the galvanic replacement reaction (GRR) method with an atomic ratio: Cu/Ag = 96.4/3.6.

Note: CuNW@AgNPs-GRR hybrids were synthesized using the following method. The as-prepared Cu NWs were evenly dispersed in 10 mL of oleylamine through sonication. Simultaneously, 20 mg of $AgNO_3$ were dissolved in 10 mL of oleylamine at 30°C through sonication. The two solutions were then mixed to initiate a galvanic replacement reaction (GRR) at 60°C for 2 hours. Afterward, the solution was cooled to room temperature, and the catalysts were washed three times with an ethanol/hexane mixture (1:1 volume ratio). The CuNW@AgNPs-GRR hybrids were collected by centrifugation at 9500 rpm for 5 minutes and dried in a vacuum oven at room temperature for 4 hours.

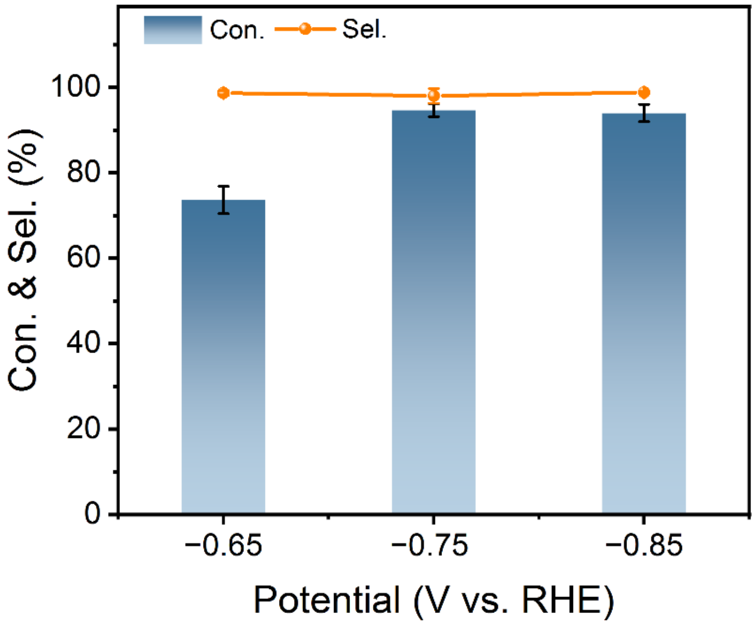

Figure S22. Potential-dependent conversion (Con.) of EYA and selectivity (Sel.) for VYA over CuNW@AgNPs-GRR bimetallic catalysts.
